# Functional and structural properties of a novel cellulosome-like multienzyme complex: efficient glycoside hydrolysis of water-insoluble 7-xylosyl-10-deacetylpaclitaxel

**DOI:** 10.1038/srep13768

**Published:** 2015-09-08

**Authors:** Tong-Yi Dou, Hong-Wei Luan, Guang-Bo Ge, Ming-Ming Dong, Han-Fa Zou, Yu-Qi He, Pan Cui, Jia-Yue Wang, Da-Cheng Hao, Shi-Lin Yang, Ling Yang

**Affiliations:** 1 Laboratory of Pharmaceutical Resource Discovery, Dalian Institute of Chemical Physics, Chinese Academy of Sciences, Dalian 116023, China; 2 University of Chinese Academy of Sciences, Beijing 100049, China; 3 Key Laboratory of Separation Sciences for Analytical Chemistry, National Chromatographic Research and Analysis Center, Dalian Institute of Chemical Physics, Chinese Academy of Sciences, Dalian 116023, China; 4 Biotechnology Institute, School of Environment and Chemical Engineering, Dalian Jiaotong University, Dalian 116028, China; 5 Jiangxi University of Traditional Chinese Medicine, Nanchang 330004, China

## Abstract

Cellulosome is a kind of multienzyme complex that displays high activity, selectivity, and stability. Here, we report a novel, non-cellulolytic, cellulosome-like multienzyme complex that produced by the *Cellulosimicrobium cellulans* wild-type strain F16 isolated from soil microflora. This multienzyme complex, with excellent catalytic efficiency of *k*_cat_ 13.2 s^−1^ to remove the C-7 xylosyl group from 7-xylosyl-10-deacetylpaclitaxel (10-DAXP), has an outstanding tolerance against organic solvents and an excellent general stability, with the long half-life of 214 hours. This cellulosome-like multienzyme complex has a novel structure distinct from the well-documented ones. The key catalytic subunit responsible for the β-xylosidase activity against 10-DAXP is identified to be a novel protein, indicating a new glycoside hydrolase (GH) family. The pioneering work described here offers a novel nanoscale biocatalyst for the production of biofuels and chemicals from renewable plant-based natural resources.

The overall performance of a biocatalyst for industrial processes depends primarily on three properties: activity, selectivity, and stability^1^. In contrast to traditional chemical catalysts, biocatalysts generally possess better activity and selectivity within the physiological range^2^. While being found to exhibit ideal activity and selectivity, lack of sufficient stability for reported biocatalysts can still be a shortcoming for their overall performance under industrial conditions^3^. Nature evolves unique strategies to combine activity, selectivity, and stability. Intracellular enzymes are located inside the cells, many of which are anchored to the membrane systems and are maintained by regeneration. For extracellular enzymes, however, multienzyme complexes, such as cellulosomes, are adopted by microorganisms as a cost-effective biosynthetic strategy^4^,^5^,^6^.

Cellulosomes are referred to as intricate multienzyme nano machines for efficient degradation of plant cell walls^4^. Typical cellulosomes are reported to be produced by some anaerobic microorganisms, e.g., the anaerobic thermophilic bacterium *Clostridium thermocellum*^5^. Cellulosomal components are assembled via a high affinity protein-protein interaction called cohesin-dockerin interaction^6^. The underlying mechanism for cellulosome’s high catalytic efficiency and overall stability are identified to be spatial proximity of the enzymes and the enzyme-substrate targeting.

*Cellulosimicrobium cellulans* is an actinobacteria, and taxonomically a typical species of the genus *Cellulosimicrobium* under the family *Promicromonosporaceae*^7^. It is known for its nitrogen fixation ability using cellulose as sole carbon source and its commercialized yeast-lytic glucanases activity^8^. Several researchers have reported cellulosome-like protuberance structures on the cell surface of *Cellulosimicrobium* and related species^9^,^10^,^11^. However, no further report of these structures has been provided.

In a project screening for glycoside hydrolases that can specifically remove the C-7 xylosyl group from 7-xylosyl-10-deacetylpaclitaxel (10-DAXP), a valuable semisynthetic precursor of the blockbuster anticancer drug paclitaxel (Taxol^®^)^12^, a wild type isolate showing the highest activity was identified to be *Cellulosimicrobium cellulans*^13^,^14^.

Here we report that the desired biocatalyst produced by a *C*. *cellulans* strain F16 is actually a nanoscale, cellulosome-like multienzyme complex. This multienzyme complex showed a catalytic rate of *k*_cat_ 13.2 s^−1^ toward 10-DAXP, a tolerance (IC_50_) of 25% against methanol, and a half-life of approximately 214 hours. Moreover, this multienzyme complex exhibited xylanase and mannase, but not cellulase activity, making it more likely to be a ‘hemicellulosome’. This report describes the isolation and characterization of such multienzyme complex, including its catalytic properties and stabilities. The basic structures of this complex are distinct from those of typical cellulosomes, because no cohesin or dockerin domains are found. The key catalytic subunit responsible for the β-xylosidase activity against 10-DAXP is identified to be a novel protein, which indicates the existence of a novel glycoside hydrolase (GH) family. Relationships between the functional attributes, i.e., catalytic activity, selectivity, and stability, and the protein structures of this complex are also investigated.

## Results

### Purification of enzymes from cell-free culture supernatant

After 24-hour incubation, culture supernatant was obtained by centrifugation using two tandem-connected centrifuges. Zymography analysis using MUX as a probe showed that there were at least five components that had β-xylosidase activity in the supernatant (Supplementary Fig. 1). These β-xylosidases were assigned as Xyl_I - Xyl_V, according to their positions (the migration rate, Rf) on the gel. According to the fluorescence intensity (grayscale analysis), Xyl_I showed approximately 74% of the total β-xylosidase activity toward MUX. As shown by further purification and identification, at least two of the five β-xylosidases, Xyl_I and Xyl_IV, were capable of removing the C-7 xylosyl group from 10-DAXP. Xyl_I was purified by a combination of ultrafiltration, ammonium sulfate precipitation, anion exchange chromatography, and gel filtration (Supplementary Fig. 2). The results showed that Xyl_I was a huge protein complex that could be trapped by the ultrafiltration membrane Biomax 500 (NMWL 500 kDa) and a 50 nm pore size ceramic tubular membrane. Such a huge particle size made the enrichment and concentration work much easier, but also made the following purification and characterization work very distinctive. Sephacryl S-1000 Superfine, a gel filtration media designed especially for the purification of nanoscale spherical particles up to 400 nm, such as plasmids, vesicles, and viruses, was used for the final, fine fractionation of Xyl_I (Supplementary Fig. 2c). This result indicated that the active component Xyl_I was actually a nanoscale particle. Xyl_I was purified with a final purification factor of 3.2 (Supplementary Table 1). Another component showing the β-xylosidase activity against 10-DAXP, designated as Xyl_IV, was partially purified from the culture supernatant with a final purification factor of 4.9 (Supplementary Fig. 2b and 3, Table 1). This “small” molecule showed an unusual “double eyelid” protein bands on the native gel, with the further migrated (higher Rf value) one exhibiting β-xylosidase activity. According to the zymography and the following purification results, approximately 95% of the β-xylosidase activity against 10-DAXP of the cell-free fermentation culture was ascribed to the nanoscale component Xyl_I (Supplementary Fig. 1).

### Purification and identification of the essential subunit

**Figure 1:**
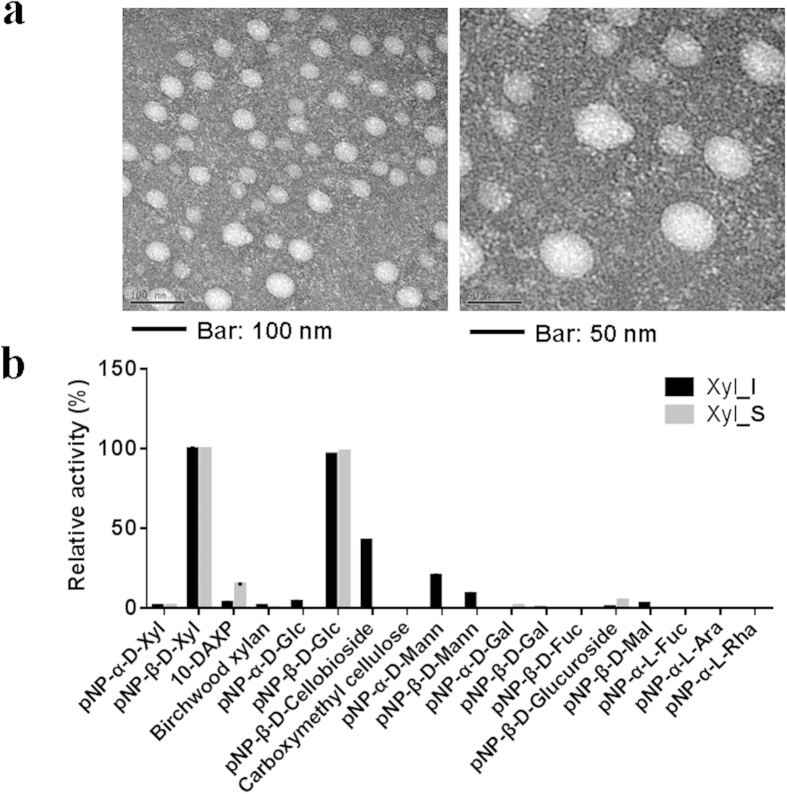
Morphology of the Purified Nanoscale Biocatalyst Xyl_I and Its Substrate Specificity. (**a**) Transmission electron microscopy analysis of the Multi protein complex that purified from the supernatant of the culture, negatively stained by phosphotungstic acid; (**b**) Substrate specificity of the purified enzymes.

The essential subunit, responsible for the 10-DAXP β-xylosidase activity of the multiprotein component Xyl_I, was isolated from the proteinase K partially digested mixture of Xyl_I and purified to homogeneity, with a purification factor of 31.8 (Supplementary Table 1, Fig. 1). This isolated subunit, i.e., Xyl_S, also showed 10-DAXP xylosidase activity. Moreover, Xyl_S also showed “double eyelid” bands on the native polyacrylamide gel with the further-migrated (higher Rf value) band to be the active form toward MUX (Supplementary Fig. 1b), which was very similar to that of Xyl_IV.

### Characterization and morphological analysis of the purified components

Dynamic light scattering (DLS) analysis showed that Xyl_I had an average diameter of 78.25 nm, and an approximate molecular weight of 17.9 MDa (Supplementary Fig. 4a). Transmission electron microscopy (TEM) analysis showed that the purified Xyl_I was an ellipsoidal or spherical nano particle, with a diameter of 20–65 nm (Fig. 1a). SDS-PAGE analysis showed that the active component Xyl_I was a multiprotein complex that composed of at least 26 subunits (see the “elucidation of the basic structures” section), further confirming that the active component Xyl_I was actually a nanoscale multiprotein complex. Based on the activity monitoring results and the electrophoresis results, it was obvious that Xyl_IV, although not purified to homogeneity, was another component responsible for the β-xylosidase activity against 10-DAXP (Supplementary Fig. 1 and 2). Electrophoresis results showed that Xyl_IV had an essential subunit of approximately 137 kDa. MALDI-TOF mass spectrometry analysis showed a molecular weight of approximately 118.5 kDa (Supplementary Fig. 4b). Meanwhile, MALDIMALDI-TOF mass spectrometry (MS) results of Xyl_S showed that the essential subunit isolated from the partially digested Xyl_I had a molecular weight of approximately 118.3 kDa (Supplementary Fig. 4c). Given the electrophoresis results (Supplementary Fig. 1), Xyl_S was probably a monomeric protein.

### Optimal conditions for the hydrolysis of 10-DAXP

**Figure 2:**
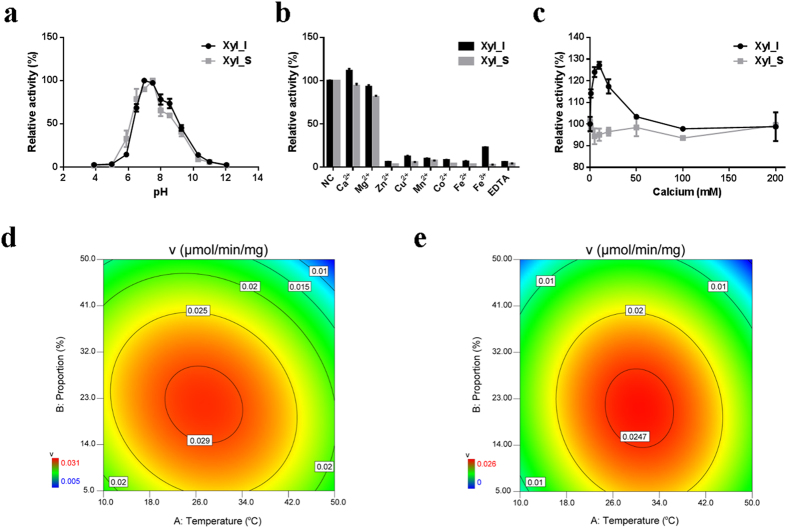
Optimum conditions for the hydrolysis of the C-7 xylosidic bond of DAXP. (**a**) Effect of pH, (**b**) metal ions and (**c**) the concentration of calcium on the 10-DAXP xylosidase activity of the purified multienzyme complex Xyl_I, and the key catalytic subunit Xyl_S isolated from it. (**d**) Xyl_I, and (**e**) Xyl_S, response surface analysis concerning the effect of reaction temperature and proportion of saturated methanol solutions on the desired activity.

To determine the optimal conditions for the hydrolysis of 10-DAXP to 10-DAP, single factors such as temperature, pH, cosolvents, and metal ions were initially investigated. The results showed that DMSO, DMF, and methanol were possible cosolvents for the above hydrolysis reaction. Considering the effects on enzyme activities, methanol was preffered for this reaction (Supplementary Table 2), particularly for the kinetic analysis of the purified enzymes against 10-DAXP. Within a short reaction time of 10–30 minutes and with methanol as the cosolvent, the optimal conditions for Xyl_I against 10-DAXP were 40 °C and pH 7.0–7.5; for the isolated subunit Xyl_S, these conditions were 30 °C and pH 7.5 (Fig. 2). Moreover, among the nine metal ions tested, only calcium showed an activation effect for the β-xylosidase activity of Xyl_I against 10-DAXP, and the maximum activity of 127% was achieved at a final Ca^2+^ concentration of 10 mM compared with the blank control (Fig. 2c). For the isolated subunit Xyl_S, however, none of the metal ions tested had activation effects on its activity against 10-DAXP (Fig. 2b). A response surface analysis using the reaction rate as the response was carried out by central composite design, taken into account the reaction temperature and the proportion of saturated methanolic solutions of 10-DAXP (8.3 mg/ml, or 8.5 mM). The results showed that, for Xyl_I, the apparent maximum reaction rate of 0.030 μmol/min/mg could be achieved at 30 °C in 25% 10-DAXP solution. For Xyl_S, this rate was 0.026 μmol/min/mg at 30 °C in 23% 10-DAXP solution (Fig. 2d,e).

### Functional parameters of the purified enzymes

**Figure 3:**
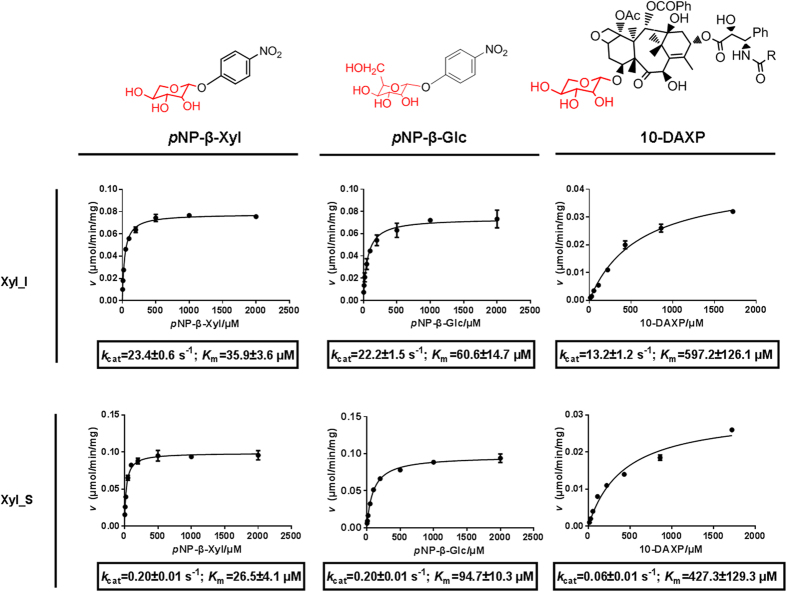
Kinetic Properties of the Purified Enzymes toward three glycosides.

Xyl_I showed an apparent *K*_m_ of 617 μM, and *k*_cat_ of 13.2 s^−1^ against 10-DAXP, whereas the apparent *K*_m_ and *k*_cat_ of Xyl_S were 410 μM and 0.061 s^−1^, respectively (Fig. 3). For the synthesized substrates *p*NP-β-Xyl and *p*NP-β-Glu, 1) both Xyl_I and Xyl_S showed smaller apparent *K*_m_ values toward *p*NP-β-Xyl than toward *p*NP-β-Glu, favoring their β-xylosidase attribute; 2) both Xyl_I and Xyl_S showed very similar *k*_cat_ value toward *p*NP-β-Xyl and *p*NP-β-Glu, indicating bifunctional β-xylosidase/β-glucosidase activity; 3) the isolated subunit Xyl_S showed a much smaller *k*_cat_ value toward either of these two substrates than Xyl_I. Moreover, Xyl_I had the best catalytic efficiency toward 10-DAXP compared to those ever been reported^13^,^15^,^16^,^17^.

### Investigation of the stabilities

**Figure 4:**
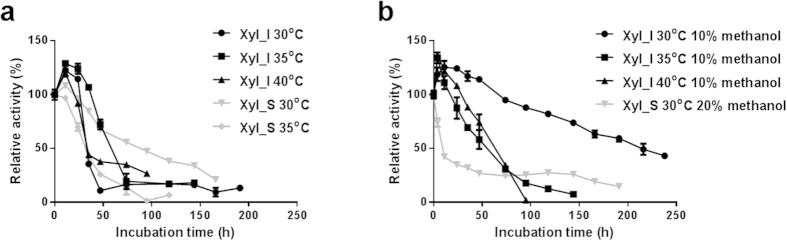
Stabilities of the Purified Enzymes toward 10-DAXP. (**a**) Thermal stability of the 10-DAXP xylosidase activity of the two purified enzymes. (**b**) stability of the 10-DAXP xylosidase activity of the two purified enzymes under reaction conditions.

To determine the overall catalytic capacity of the purified enzymes, their stabilities were also investigated. At 30 °C and 10% methanol, the half-life of Xyl_I was approximately 214 hours, in sharp contrast with only 6 hours for the isolated subunit Xyl_S (Fig. 4).

### Whole genome shotgun (WGS) sequencing

Using second generation high-throughput sequencing technology, 606 Mb of sequencing reads were generated, corresponding to approximately 130-fold genome coverage. These short reads were assembled into 15 contigs, and subsequent 14 scaffolds. In total, this unclosed draft genome sequence of the *Cellulosimicrobium cellulans* strain F16 showed a size of 4,602,907 bp, with a mean GC content of 74.4%. According to gene prediction and annotations of NCBI PGAP pipeline, 4,155 genes were found, of which 4,102 were protein coding sequences (CDS), and 51 were tRNA genes. The full length gene sequences of 16S and 23S rRNA were also found. This WGS project has been deposited at DDBJ/EMBL/GenBank under the accession ATNL00000000. The version described in this paper is version ATNL01000000. The corresponding BioProject number is PRJNA209578 (Supplementary Table 3). The translated protein sequences were used as a database for the following proteomic analysis.

### Elucidation of the basic structures

**Figure 5:**
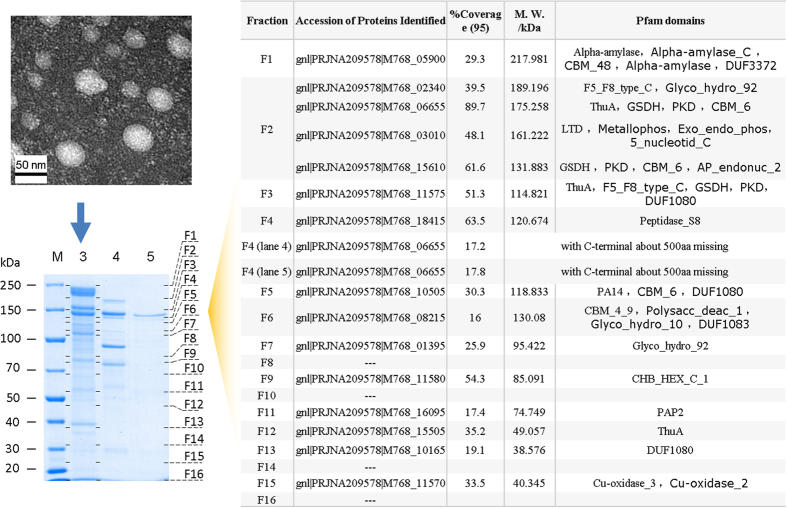
Basic Structures of the Purified multienzyme complex Xyl_I.

**Figure 6:**
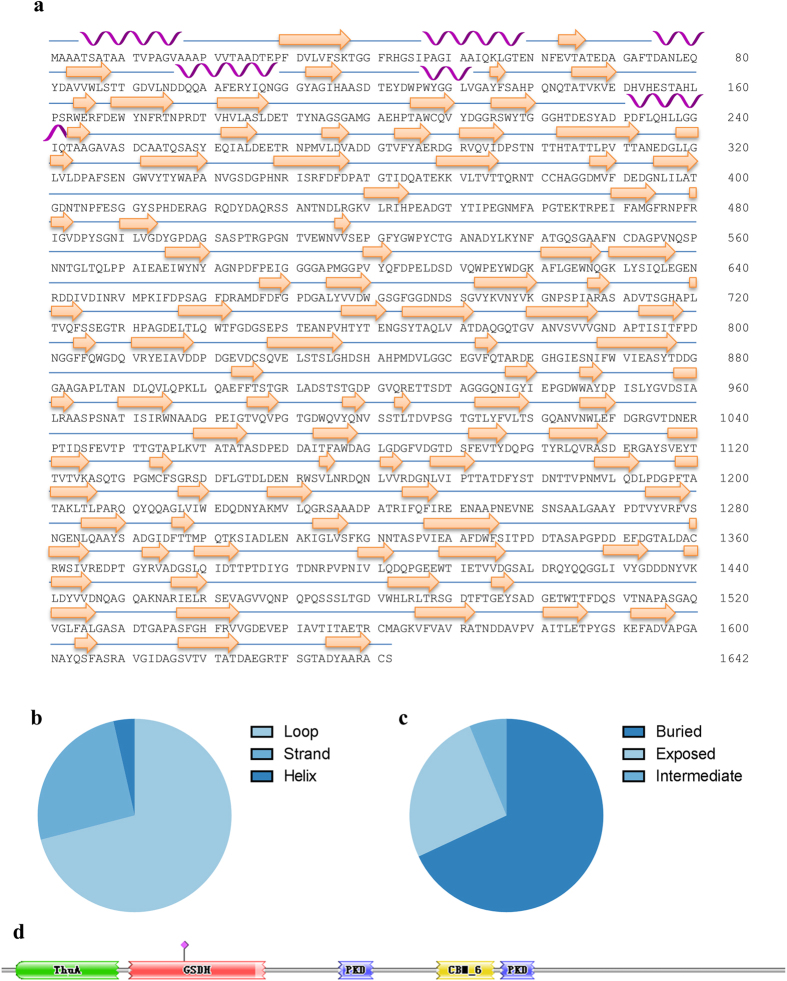
Basic Structures of the Key Subunit: the Hypothetical Protein M768_06655. (**a**) Primary structure and the corresponding secondary structure predicted by PredictProtein, cartoons were drawn manually according to the prediction, helix—alpha helix, arrow—beta sheet, line-loop structures. (**b**) and (**c**), pie chart showing predicted secondary structure composition and solvent accessibility; (**d**) Protein families.

To determine the basic structures of these enzymes, three purified enzymes, Xyl_I, Xyl_IV, and Xyl_S, were initially analyzed by SDS-PAGE. The results showed that Xyl_I was actually an enormous multiprotein complex composed of at least 26 subunits, whereas both Xyl_IV and the purified subunit Xyl_S were identified to be a 130 kDa subunit (Fig. 5). Combined with the MALDI-TOF results (Supplementary Fig. 4b,c), these data suggest that these two enzymes are monomeric proteins. To further reveal their primary structures, Xyl_I and Xyl_S were digested by trypsin directly, and then analyzed using the AB SCIEX TripleTOF^®^ 5600 system. The partially purified component Xyl_IV was firstly separated by an 8% native-PAGE gel. The active band, shown by in gel staining using a synthesized fluorescent probe MUX, was cut, in-gel digested by trypsin, and analyzed using the same LC-MS/MS system. The data were then searched against the translated protein sequences from the WGS data. The results showed that, 1) 27 proteins were identified to be subunits of the nanoscale biocatalyst Xyl_I. Further functional predictions showed that they are glucanases, xylanases, mannases, peptidases, and laccases, i.e., mostly hemicellulases. 2) Interestingly, both Xyl_S and the cut-from-gel component Xyl_IV were identified as the hypothetical protein M768_06655, with the C-terminal ~500 aa missing (Fig. 5). However, the full-length version of this protein could be found from the proteomic results of Xyl_I, with a high coverage of 89.7%. Protein domain and family analysis by Pfam^18^ showed that this hypothetical protein had five significant Pfam-A matches: ThuA (PF06283), GSDH (PF07995), PKD (PF00801), CBM_6 (PF03422), and PKD (PF00801), from the N terminus to the C terminus (Fig. 6d). For further verification and identification of the subunits comprising Xyl_I, the protein bands separated by 10% SDS-PAGE were cut from the gel, in-gel digested by trypsin, analyzed by LTQ LC-MS/MS, and then searched against the translated genome database. The results showed that the hypothetical protein M768_06655 was at the position of ~200 kDa on the gel (Fig. 5). This result validated that Xyl_I contains a full length version of this protein, and both that Xyl_IV and Xyl_S were truncated versions, with the C-terminal approximately 500 aa missing.

### Prediction of the secondary structure and solvent accessibility of the hypothetical protein M768_06655

The predicted results given by the online server PredictProtein^19^ showed that the hypothetical protein M768_06655 was composed of 3.9% helix structure, 31.2% strand, and an unusually high amount of loop regions of 64.9%. This result indicates a high conformational flexibility and a compact packing density (Fig. 6, Supplementary Information II). The predicted solvent accessibility confirms such a compact folding. Moreover, several amino acid residues were predicted to be involved in external protein-protein interactions.

## Discussion

10-DAXP is one of the most valuable natural derivatives of paclitaxel (Taxol^®^)^12^, especially in endemic *Taxus* species in China. Its content can reach up to 0.1% in the needles and branches of *T. yunnanensis* and *T. mairei*, two endemic *Taxus* species that are extensively cultivated in China^20^. The aglycone 10-DAP can be transformed into paclitaxel simply by C-10 acetylation, which makes ‘10-DAXP→10-DAP→paclitaxel’ a value-added route for further ameliorating the paclitaxel supply crisis^21^. However, it appears that the β-xylosidic bond of 10-DAXP is somehow special, and obtaining a biocatalyst that can specifically remove the C-7 xylosyl group from 10-DAXP has been challenging^15^. To date, only *Moraxella sp*^15^., *Leifsonia shinshuensis*^13^, *Enterobacter sp*^16^., *Streptomyces matensi*, *Arthrobacter nicotianae*, *Achromobacter piechaudii*, and *Pseudomonas plecoglossicida*^17^, and *Lentinala edodes*^22^ have been found to have such specific activity. Even so, their activity and stability still cannot meet the demand for the industrial transformation of 10-DAXP into 10-DAP.

Compared with other glycoside compounds, 10-DAXP is unique because: it has a much larger and lyophobic aglycone group that requires organic solvents to increase its final concentration in the reaction system; and a complicated and relatively unstable parent nucleus, specifically an unstable chiral structure at C-7 of the product (10-DAP). Correspondingly, the catalyst required should have (1) good catalytic efficiency (*k*_cat_/*K*_m_) against 10-DAXP under moderate conditions, (2) excellent selectivity to specifically (ideally 100%) remove the C-7 xylosyl group while maintaining the structure of the aglycone, (3) good tolerance against organic solvents such as methanol, and (4) acceptable general stability. In addition, it will be more ideal for industrial processes if this catalyst is cheap and easy to obtain.

This paper reports an extracellular, nanoscale multienzyme complex Xyl_I produced by the *Cellulosimicrobium cellulans* wild-type strain F16. This biocatalyst can specifically remove the C-7 xylosyl group from 10-DAXP with the highest catalytic activity to date^13^,^15^,^16^,^17^,^22^. In addition, it has excellent stability under the reaction conditions, presenting a theoretical total catalytic capacity of approximately 10^7^ reaction cycles. Moreover, its nanoscale properties make it easy to be enriched and recovered by ultrafiltration, highlighting its great potential for the industrial production of biofuels and chemicals from renewable plant-based natural resources.

It should also be noted that, given its multienzyme activity, nanoscale size and need for calcium to retain activity, the active component Xyl_I is very similar to the well-documented multienzyme complex, the cellulosome^4^,^5^,^6^. However, Xyl_I produced by strain F16 is vastly different from classic cellulosomes in that 1) strain F16 was able to digest microcrystalline cellulose, but no CMCase activity was detected in the cell-free culture supernatant, and 2) none of the molecular features of typical cellulosomes, such as the dockerin or cohesin domains, were found in the subunits of Xyl_I. Indeed, no dockerin or cohesin domains were found throughout the translated genome data. Therefore, it is more appropriate to refer to Xyl_I as the hemicellulosome, a cellulosome-like multienzyme complex that could degrade hemicellulose but without cellulolytic activity. The hemicelluloses (e.g., those found in corn cob) are digested in sequential catalytic transformations by the hemicellulasesof Xyl_I, such as xylanases, glucanases, and mannases. Several researchers reported cellulosome-like protuberance structures on the cell surface of *Cellulosimicrobium* and related species^9^,^10^,^11^. However, no further work of these structures has ever been presented. The work reported here, for the first time, provides experimental evidence that the wild-type *C. cellulans* strain F16 (aerobic and facultative anaerobic) produces a cellulosome-like, nanoscale multienzyme complex when grown on polysaccharides such as corn cob or cellulose.

**Figure 7:**
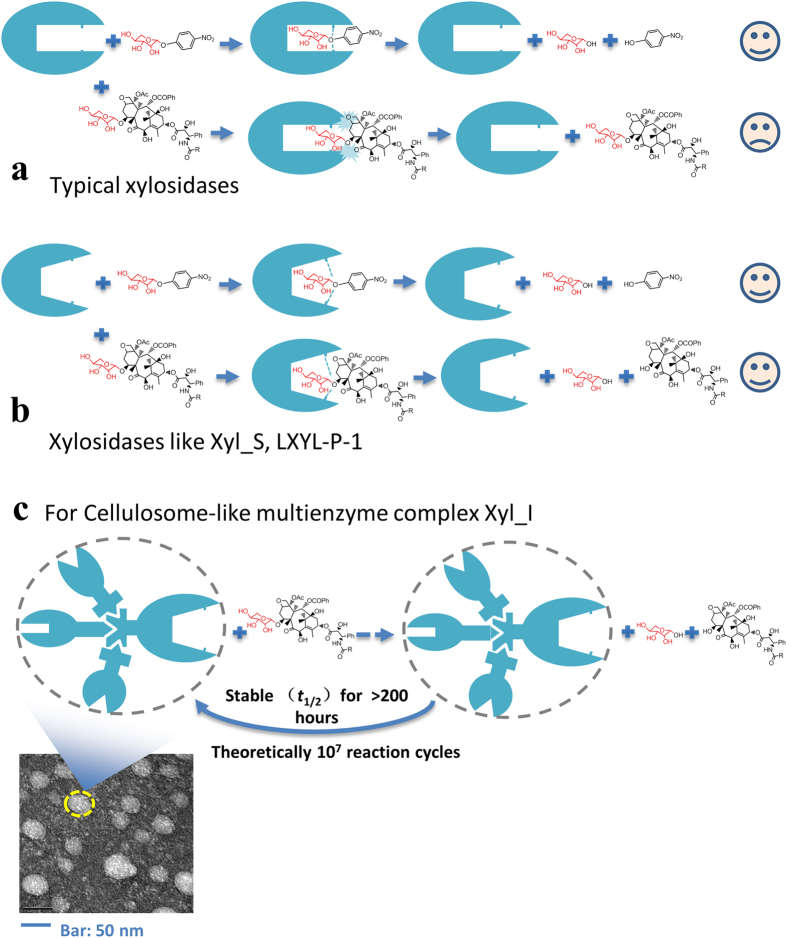
Illustrations Showing Possible Structure-Function Relationships of the Novel Biocatalyst Xyl_I. (**a**) Illustrations showing presumed structural features that typical xylosidases unable to remove the C-7 xylosyl group from 10-DAXP; (**b**) presumed structural features that xylosidases like Xyl_S, or LXYL-P-1 capable of removing the C-7 xylosyl group from 10-DAXP; (**c**) presumed structural features of the stability and the nanoscale multienzyme properties of the cellulosome-like biocatalyst Xyl_I.

Previous studies have shown that ordinary β-xylosidases did not show any activity toward 10-DAXP^15^. The critical reason might be the intrinsic features of the molecular structure of 10-DAXP, i.e., a much larger and hydrophobic aglycone group on the other side of the glycosidic bond. Furthermore, exoglycosidases, including β-glucosidase, β-xylosidase, and β-galactosidase, generally have ‘pocket’ or ‘crater’ topology^23^. Thus, for ordinary β-xylosidases, the steric hindrance of the aglycone group might block the C-7 β-xylosidic bond of 10-DAXP from reaching the catalytic site inside the ‘pocket’, ultimately preventing it from being hydrolyzed (Fig. 7a). Therefore, β-xylosidases that could remove the C-7 xylosyl group from 10-DAXP should have a novel catalytic center, such as a broader or more flexible ‘pocket’ or ‘crater’ (Fig. 7b). The enzymes that were identified by Cheng *et al.*^22^, namely LXYL-P-1 and LXYL-P-2, seem to support this inference. These two enzymes showed a novel primary sequence, and were classified with a considerably low amino acid sequence similarity into the GH family 3, a group of enzymes that in many cases have dual or broad substrate specificities^24^,^25^. They were also found to have bifunctional β-xylosidase/β-glucosidase activity, which indicates a broader or more flexible catalytic center. Interestingly, the key subunit of the biocatalyst Xyl_I, the hypothetical protein M768_06655, was also novel, which does not belong to any known GH families, and shows a very low sequence identity (<10%) to that of LXYL-P-1 and −2. Although five protein domains of M768_06655 were identified by Pfam, it is eclusive which one is the catalytic center responsible for the β-xylosidase activity against 10-DAXP. However, similar to LXYL-P-1 and −2, the hypothetical protein M768_06655 showed bifunctional β-xylosidase/β-glucosidase activity which, to some extent, supports the above hypothesis. It seems that there are intricate correlations between 10-DAXP β-xylosidase activity and bifunctional β-xylosidase/β-glucosidase activity. As discussed above, these bifunctional enzymes may have a broader or more flexible catalytic center to accommodate the aglycone of 10-DAXP, so that its C-7 β-xylosidic bond could reach the catalytic site and then be hydrolyzed (Fig. 7b).

It should also be highlighted that the assembled intact Xyl_I shows a much higher catalytic rate than the isolated subunit Xyl_S. Like typical cellulosomes, the synergistic effects of the Xyl_I subunits facilitate the catalytic efficiency^6^. However, many other factors may also contribute to such differences. For instance, one complex of Xyl_I may have more than one M768_06655 proteins, or the catalytic rate of the key subunit may be compromised due to the deletion of the C-terminal 500 aa during the partial digestion by proteinase K. These possible causes warrant further studies.

The predicted secondary structure and solvent accessibility results indicated that M768_06655 might have a compact folding structure, which may explain its excellent methanol tolerance. However, the intact multienzyme complex Xyl_I has a much better general stability. Like typical cellulosomes, the spatial proximity effects between the subunits might protect them from being attacked^4^,^5^,^6^. Moreover, the multienzyme complex reported here requires calcium to retain its activity, while no activation by calcium was detected for the isolated subunit Xyl_S. This result may indicate that calcium was probably used to stabilize the quaternary structure of the intact multienzyme complex. This stabilization may occur, for instance, by enhancing the interactions between the subunits^26^,^27^ (Fig. 7c), although it is still not clear how these subunits interacted with each other to form Xyl_I.

In summary, this paper reports a cellulosome-like nanoscale multienzyme complex Xyl_I that has not only excellent potential for industrial applications but also great academic significance. Xyl_I may possess a novel structure for protein-protein interactions. The key subunit responsible for the β-xylosidase activity against 10-DAXP, designated as Xyl_S, is a novel protein and perhaps represents a novel GH family as well. However, it is still ambiguous 1) how these subunits are assembled to form such a nanoscale entity, 2) what the relationship is between the active components, Xyl_I and Xyl_IV, that existed in the cell-free culture supernatant, 3) why strain F16 produces such a multienzyme complex. Nevertheless, these questions do not hinder us from coming to the following conclusions: 1) this biocatalyst reported here may offer a powerful tool for the efficient hydrolysis of β-xylosides and β-glucosides with large steric hindrance aglycones, such as 10-DAXP; 2) when developing more ideal biocatalysts, cellulosomes or cellulosome-like multienzyme complexes might be an effective strategy for combining activity, selectivity, and stability. Such a multienzyme complex is even comparable to the popular immobilized enzymes^28^. For instance, cellulosomes are now being studied extensively for use in biomass conversion to biofuels^4^,^27^.

## Methods

### Reagents

Samples of the 7-xylosyltaxanes mixtures, containing 55% 10-DAXP, were purchased from Jiangsu Yew Pharmaceutical Co., Ltd, China. The standard sample of 10-DAXP was prepared in this lab. All the synthetic glycosides used here were purchased from Sigma-Aldrich (St. Louis, MO). The other chemicals used were analytical reagent grade.

### Fermentation and enzyme production

Corn cob of 1.4% was used as the sole carbon source in the culture medium. For enzyme production, *Cellulosimicrobium Cellulans* strain F16 (CCTCC patent strain No. M 2013201)^14^ was grown in complex medium, containing 1.4% corn cob, peptone (0.4%), yeast extract (1%), and K_2_HPO_4_•3H_2_O (0.4%). Cells were firstly grown in a 250 ml flask containing 50 ml culture medium, and then transferred to a 50-liter in-situ sterilization fermenter containing 35 liters of the medium. Enzyme production was finally performed in a 500-liter in-situ sterilization fermenter containing 350 liters of the medium. The whole fermentation process was kept at 30 °C for 24 hours.

### Purification of the active components from the cell-free culture supernatant

The overall purification strategy was shown in the flow chart (Supplementary Fig. 3). Culture supernatant was obtained by centrifugation using two tandem-connected centrifuges. For purification and further studies, 55 liters of the supernatant were disposed in an industrial ultrafiltration system with a ceramic tubular membrane of 50 nm pore size. A two-step ammonium sulfate precipitation (first step 0–20% saturation; second step 20%–40% saturation) was then used to partially purify the active components from the ultrafiltration retentate. Anion-exchange chromatography of the 20%–40% ammonium sulfate precipitation fraction was carried out with a Source 15Q 5/50 column (GE Healthcare Life Sciences, laboratory packed). Fractions of 2 ml each were collected. Fractions F10–F14 containing the active component Xyl_I were pooled, concentrated by ultrafiltration, and further fractionated by gel filtration using a Sephacryl S-1000 SF 17/700 column equilibrated with 50 mM Tris-HCl buffer, pH 7.5, containing 0.15 M NaCl. Fractions F8–F9 containing the active component Xyl_IV were also pooled, concentrated by ultrafiltration, and applied to a Sephacryl S-200 HR 17/750 column equilibrated with the same buffer used for Sephacryl S-1000. The key catalytic subunit of the multiprotein complex Xyl_I was isolated as follows: Partial digestion was performed following the procedure of Ely Morag *et al.* with minor modifications^29^. Proteinase K (0.60 mg/ml) was mixed with the purified Xyl_I (14.4 mg/ml), and then incubated for 4 hours at 37 °C in 50 mM Tris-HCl buffer (pH 7.5). The partially digested multiprotein complex was then loaded to a Sephacryl S-200 HR 17/755 column to separate the released subunits. Active fractions were pooled, and then loaded onto a Source 15Q 5/50 column equilibrated with 50 mM Tris-HCl buffer, pH 7.5, for further purification. Fractions active to 10-DAXP was pooled, and used for further research.

### Electrophoresis and zymography

Non-denaturing PAGE was performed on 2%–15% continuous gradient polyacrylamide gels without spacer gel. MUX is a synthesized fluorescent probe for detection of the β-D-xylosidase activity. After electrophoresis, gels were put into a 50 mM pH 7.5 Tris-HCl buffers containing 0.5 mM MUX and 10 mM CaCl_2_, and incubated at 30 ^o^C for 5 minutes. Then gels were viewed and photographed under ultraviolet (UV, 365 nm), and finally restained again with Coomassie Brilliant Blue R-250. SDS-PAGE was performed on 10% polyacrylamide gels.

### Size and molecular weight

The size and molecular weight of the purified Xyl_I were analyzed by a Zetasizer Nano S90 (Malvern Instruments Ltd., U.K.) by the DLS technology. The transmission electron microscope (TEM) analysis of the purified multienzyme complex Xyl_I was performed by negative staining using 2% aqueous phosphotungstic acid on a forvad membrane mesh^30^,^31^. A JEOL JEM-2100F TEM was used. The exact molecular weight of Xyl_IV and Xyl_S was analyzed by the AB SCIEX TOF/TOF™ 5800 System.

### Determination of the optimum conditions for biotransformation of 10-DAXP to 10-DAP

The optimal pH assay was conducted with a fixed temperature of 30 °C, and a pH range of 5.0 to 11.0. Determining the optimal temperature of the enzyme samples against 10-DAXP was performed using 5 mg/ml 10-DAXP dissolved in methanol. To determine the effect of metal ions (Ca^2+^, Mg^2+^, Zn^2+^, Cu^2+^, Mn^2+^, Co^2+^, Fe^2+^, Fe^3+^) and EDTA on the enzyme activity, enzymes were properly diluted with 50 mM Tris-HCl (pH 7.5) containing 1 mM of CaCl_2_, MgCl_2_, ZnCl_2_, CuCl_2_, MnCl_2_, CoCl_2_, FeCl_2_, FeCl_3_, or 0.1 mM EDTA, mixed with 10 μl 10-DAXP (dissolved in methanol, 5 mg/ml), and incubated at 30 °C for 30 min. The release of 10-DAP was measured as described above. The enzyme solution without metal ions or EDTA was used as control and the activity was designated as 100%. The effect of organic solvents on enzyme activity was also determined similarly as described above. Thirteen organic solvents with Log*P*_o/w_ value ranging from −1.35 to 4.5 were tested (Supplementary Table 2). Proportions that inhibit 10% and one half of the original activity were determined, and shown as the IC_10_ and IC_50_ value of one specific organic solvent. All of the tests were performed in duplicate or triplicate.

### Enzyme activity assay and substrate specificity

Assays were performed in a total volume of 200 μl in 50 mM Tris-HCl buffer, pH 7.5, containing 5–30 μl of the enzyme solution, 10 μl CaCl_2_ (200 mM, dissolved in distilled water) and 10 μl 55% 10-DAXP (dissolved in methanol, 5 mg/ml), and incubated at 30 °C for 10–30 minutes. Reaction mixtures were analyzed by an HPLC-UV method on a Shimadzu LC high-performance liquid chromatography system, with a Kromasil C18 5 μ, 150 mm × 4.6 mm column. The UV detector was set at a wavelength of 227 nm. One unit of the enzyme activity was defined as the amount of enzymes that can release 1 μmol 10-DAP per minute, in 50 mM Tris-HCl buffer, pH 7.5, at 30 °C. To study the glycoside specificity of the enzymes, 15 synthesized *p*-nitrophenyl glycosides (Sigma) were used as substrates. Reactions were performed at 30 °C with shaking in a Synergy™ H1 multi-mode plate reader (BioTek, USA). Absorbance at 405 nm was monitored every minute for totally 30 minutes. The initial slope of the time course was used as the reaction velocity. The CMCase activity and xylanase activity were determined by the DNS method^32^. Reactions were terminated by adding 0.6 ml DNS solution and incubated at 99 °C for 5 min, then measured for reducing sugar at 540 nm.

### Determination of the catalytic efficiency

Determination of the kinetic parameters (apparent *V*_max_ and *K*_m_) toward synthetic glycoside *p*NP-β-D-Xyl and *p*NP-β-D-Glc were performed using the same multi-mode plate reader described above. The substrate concentration range of 0.005–2.000 mM (stock solution: 20 mM each dissolved in distilled water). The time courses of each reaction were monitored every 30 seconds under the wavelength of 405 nm. Each velocity was generated within the initial linear region of the time course. The kinetic parameters toward 10-DAXP were determined with the 10-DAXP concentration range of 0.010–1.74 mM (10-DAXP stock solution: 8.2 mg/ml, 8.6 mM, dissolved in methanol). All the kinetic data were processed using GraphPad Prism software (GraphPad, San Diego, CA, USA) by nonlinear regression (curve fit), based on Michaelis-Menten enzyme kinetics.

### Investigation of the general stability

For the investigation of the enzyme’s thermal stability, six tubes of enzyme samples, 1.5 ml each, were incubated separately at 30 °C, 35 °C, or 40 °C. For the investigation of the enzyme stability at reaction conditions (with methanol, without 10-DAXP), 1.5 ml enzyme samples were incubated separately in the existence of methanol at the IC_10_ point. Enzymatic activity toward 10-DAXP was detected every 12–24 hours until it dropped to less than half of the initial activity. The enzyme activities at 0 hours were designated as the initial activity 100%.

### Whole-genome shotgun (WGS) DNA sequencing

The genome of the wild-type strain F16 was sequenced to a fine scale. WGS DNA sequencing was performed by BGI (Beijing Genomics Institute, Shenzhen) using Illumina HiSeq 2000 sequencing platform. The WGS sequence data was then assembled into contigs using SOAPdenovo v.1.04^33^. Gene prediction and annotation was performed by the NCBI Prokaryotic Genome Annotation Pipeline (released 2013, http://www.ncbi.nlm.nih.gov/genome/annotation_prok/), with the Annotation Software revision 2.1 (rev. 407375). CAZy database and CAZymes Analysis Toolkit (CAT), with the sequence based annotation choice (see http://mothra.ornl.gov/cgi-bin/cat/cat.cgi for more details)^34^ were also used to annotate the newly sequenced genome.

### Protein identification by LC-MS/MS

The fully dissociated subunit mixtures of the purfied multienzyme complex Xyl_I were digested by trypsin as reported by Wang *et al.*^35^. For proteomic analysis, Xyl_I was firstly resolved by SDS-PAGE, and then the subunits were further identified by cutting the protein band(s) from the gel, digested by trypsin, and analyzed by proteomic LC-MS/MS. The purified subunit Xyl_S was digested by trypsin directly. For partially purified Xyl_IV, the subunit predicted to be responsible for the 10-DAXP glycoside hydrolase activity was cut from gel, and digested by trypsin according to an in-gel digestion protocol^36^. A Waters NanoACQUITY UPLC system was used to deliver the mobile phase. Tandem mass spectrometry was performed on a Triple TOF 5600 mass spectrometer (AB SCIEX, USA) Identification of peptides and proteins was performed using the Paragon Algorithm in ProteinPilot^TM^ 3.0 software (AB SCIEX, USA) with trypsin specificity in “Thorough ID” mode. The results of these three samples were searched against the protein sequences predicted from the WGS data of *C*. *cellulans* F16 (GenBank accession: ATNL00000000). The software Armone was applied to validate the identifications as reported by Jiang *et al.*^37^. Herein, Rank’m, ΔCn’m, and Xcorr’s were used as cut off filters to achieve FDR < 1% at the peptide level. Functions of the identified proteins were predicted by searching against the protein domain database Pfam^18^.

### Prediction of the secondary structure

Prediction and analysis of the secondary structure, solvent accessibility, and protein-protein binding sites of the hypothetical protein M768_06655 were performed using an integrated open source online prediction tool PredictProtein^38^ (https://www.predictprotein.org/).

## Additional Information

**How to cite this article**: Dou, T.-Y. *et al.* Functional and structural properties of a novel cellulosome-like multienzyme complex: efficient glycoside hydrolysis of water-insoluble 7-xylosyl-10-deacetylpaclitaxel. *Sci. Rep.* **5**, 13768; doi: 10.1038/srep13768 (2015).

## Supplementary Material

Supplementary Information
