# Supplementary material for: Functional and structural properties of a novel cellulosome-like multienzyme complex: efficient glycoside hydrolysis of water-insoluble 7-xylosyl-10-deacetylpaclitaxel
Source: Sci Rep. 2015 Sep 8;5:13768. doi: 10.1038/srep13768 (PMC4562250; doi:10.1038/srep13768)
Supplement: Supplementary Information [file srep13768-s1.pdf]

Manuscript title: Functional and Structural Properties of A Novel Cellulosome-like Multienzyme Complex: Efficient Glycoside Hydrolysis of Water-insoluble 7-Xylosyl-10-deacetylpaclitaxel

by: Tong-Yi Dou, Hong-Wei Luan, Guang-Bo Ge, Ming-Ming Dong, Han-Fa Zou, Yu-Qi He, Pan Cui, Jia-Yue Wang, Da-Cheng Hao, Shi-Lin Yang, Ling Yang*

Supplementary Information

Figure 1 Electrophoresis analysis of the purified enzymes

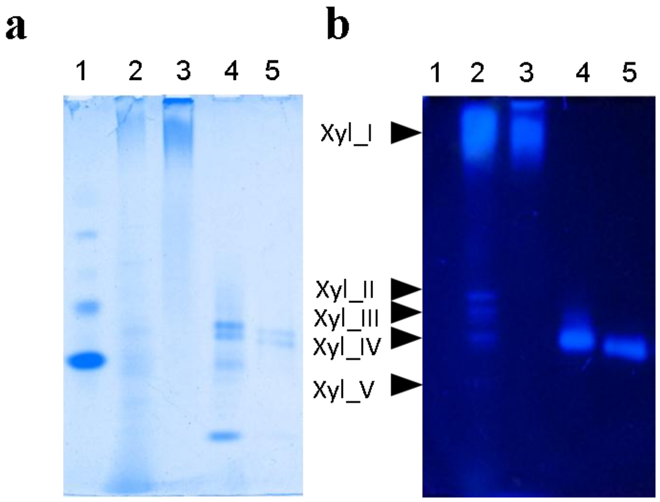

(**a**) 2%-15% continuous gradient Native PAGE, coomassie blue staining; (**b**) Zymogram (MUX in situ staining) of (**a**); 1-BSA, BSA dimer, ferritin; 2-cell-free culture supernatant; 3-the purified multienzyme complex Xyl_I; 4-partly purified active component Xyl_IV; 5-the purified key catalytic subunit Xyl_S

Figure 2 Chromatography for the isolation of active components

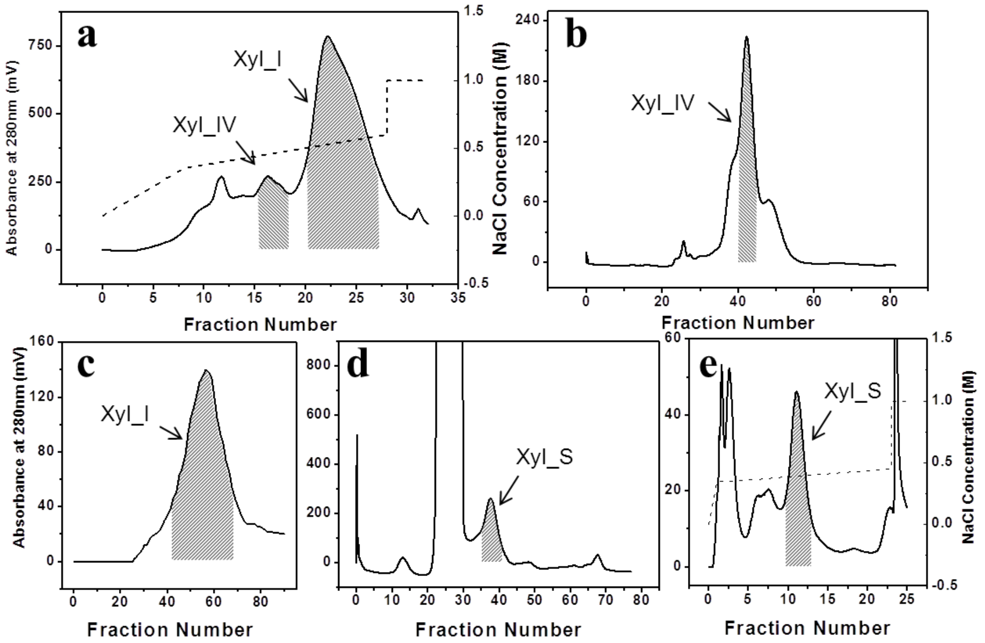

(**a**) Ion Exchange Chromatography by Source 15Q 11/80, Sample: Ammonium Sulfate Precipitation Fraction 20%-40% (**b**) Gel Filtration Chromatography by Sephacryl S-200 HR 17/750, Fractions containing Xyl_IV; (**c**) Gel Filtration Chromatography by Sephacryl S-1000 SF, Fractions containing Xyl_I; (**d**) Gel Filtration Chromatography by Sephacryl S-200 HR, Sample: partial digestion mixture of Xyl_I; (**e**) Ion Exchange Chromatography by Source 15Q 5/50, Sample: Fractions containing Xyl_S

Figure 3 Flow Chart of the overall purification strategy performed in this work

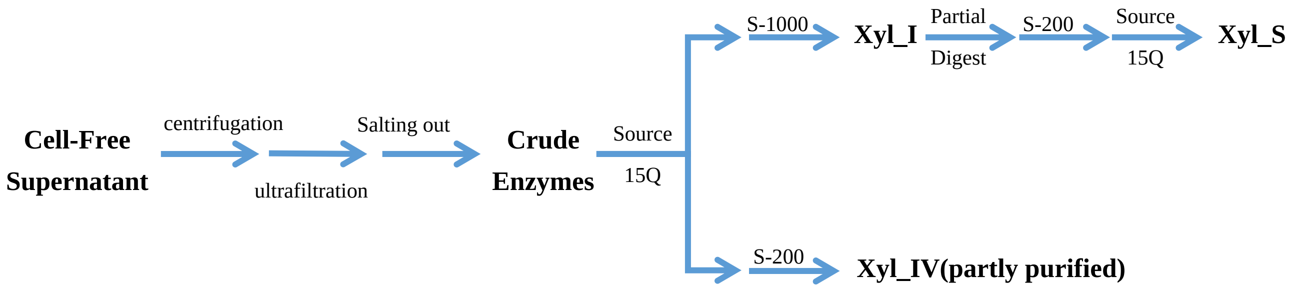

Figure 4 Molecule Size and Morphology Analysis of the Purified Enzymes

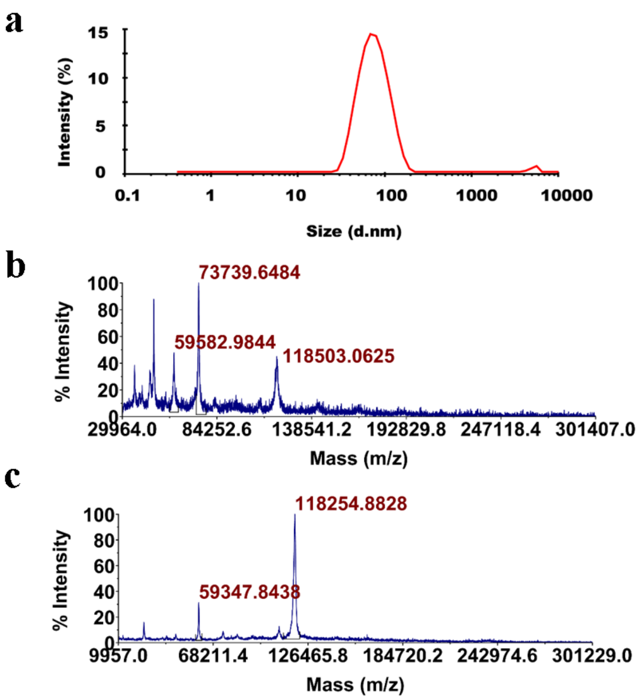

(**a**) Particle size distribution analysis of the purified multienzyme complex Xyl_I, (**b**) Maldi-TOF analysis of the partly purified active component Xyl_IV, (**c**) Maldi-TOF analysis of the purified subunit Xyl_S

Table 1 Purification Summary

Sample	Total Volume /ml	Total Protein /mg	Total Activity /U	Specific Activity /mU/mg	Yield /%	Purification Fold
culture Supernatant	13750	26538	129.3	4.9	100	1
Ultrafiltration	2000	9720	106.6	11	82.4	2.3
Ammonium Sulfate precipitation 20%-40%	110	1487	17.5	11.8	13.5	2.4
Source 15Q (Xyl_I)	1100	836	12.3	14.7	9.5	3
Sephacryl S-1000 SF	1680	605	9.6	15.8	7.4	3.2
Source 15Q (Xyl_IV)	16	9.8	0.016	1.7	100	1
Sephacryl S-200 HR	2	0.1	0.001	8.3	6.1	4.9
Partial digestion mixture of Xyl_I	2	28.8				
Sephacryl S-200 HR	10					
Source 15Q (Xyl_S)	20	3	0.5	154.8	-	31.8

Table 2 Organic Solvent Tolerance

Organic solvent	Log$P_{o/w}$	IC_{10} (%)		IC_{50} (%)		10-DAXP solubility at IC_{10} (mg/ml)	
		Xyl_I	Xyl_S	Xyl_I	Xyl_S	for Xyl_I	for Xyl_S
water						0. 02±0. 00	
DMSO	-1.35	3.5	7.5	17.9±2.4	32.1±3.7	0.68±0. 04	1.38±0. 03
DMF	-1	3.5	9	18.7±6.0	34.4±4.7	0.71±0. 02	1.51±0. 01
Methanol	-0.76	9	20	25.0±1.4	42.8±3.4	0.83±0. 01	1.60±0. 01
Ethanol	-0.24	13	9	21.5±3.9	36.9±6.7	0. 15±0. 00	0.09±0. 00
Acetone	-0.23	3	3.7	12.7±1.9	16.8±1.7	N.D.	N.D.
Ethyl acetate	0.73	13.3	8	24.1	36.5±2.6	0.02±0. 00	0.02±0. 00
1-Butanol	0.8	11	2.4	18.1±3.6	24.2	N.D.	N.D.
Benzene	2	6	1.9	30.7±5.0	46.6±4.7	N.D.	N.D.
Chloroform	2	11	1.5	45.6±7.6	>100	N.D.	N.D.
Octanol	3.15	7.5	2	21.1±1.7	76.8±8.0	N.D.	N.D.
n-Hexane	3.5	>100	>100	17.9±2.4	32.1±3.7	N.D.	N.D.
n-Heptane	3.5	>100	>100	18.7±6.0	34.4±4.7	N.D.	N.D.
n-Octane	4.5	>100	>100	25.0±1.4	42.8±3.4	N.D.	N.D.

Table 3 Genome statistics

Statistics	
Num of Scaffold	14
Total Length(bp)	4602907
N50	666262
N90	244600
Max Length(bp)	1462680
Max Length(bp)	515
G+C Content(%)	74.4
Predicted Coding Sequences	4102
tRNAs	51
rRNA operons	2 (16S, 23S)
GenBank Accession	ATNL00000000
Bioproject	PRJNA209578

Table 4 Proposed Subunits of Xyl_I Identified by AB SCIEX Triple-TOF 5600

N	Accession of Proteins Identified	%Coverage(95)	Specific Peptide Identified	Molecular Weight/kDa	CAZy Families	Pfam domains	signal peptide detected	Location
1	gnl|PRJNA209578|M768_06655	89.7	1389	175.258	CBM6	ThuA，GSDH，PKD ，CBM_6	Sec	Scaffold2_1
2	gnl|PRJNA209578|M768_15610	61.6	135	131.883	CBM6	GSDH ，PKD ，CBM_6 ，AP_endonuc_2	Sec	Scaffold6_1
3	gnl|PRJNA209578|M768_18415	63.5	145	120.674	GH43 CBM22	Peptidase_S8	Sec	Scaffold9_1
4	gnl|PRJNA209578|M768_11575	51.3	101	114.821	CBM32	ThuA，F5_F8_type_C，GSDH，PKD，DUF1080	Sec	Scaffold3_1
5	gnl|PRJNA209578|M768_03010	48.1	99	161.222	CBM5	LTD ，Metallophos，Exo_endo_phos，5_nucleotid_C	Sec	Scaffold1_1
6	gnl|PRJNA209578|M768_02340	39.5	107	189.196	GH92	F5_F8_type_C ，Glyco_hydro_92	Tat	Scaffold1_1
7	gnl|PRJNA209578|M768_11580	54.3	93	85.091	CBM6	CHB_HEX_C_1	Sec	Scaffold3_1
8	gnl|PRJNA209578|M768_05900	29.3	46	217.981	CBM48 GH13	Alpha-amylase，Alpha-amylase_C ，CBM_48 ，Alpha-amylase ，DUF3372	Tat	Scaffold1_1
9	gnl|PRJNA209578|M768_11760	58.5	52	59.25	CBM12	PA ，Peptidase_M28	Sec	Scaffold3_1
10	gnl|PRJNA209578|M768_10505	30.3	30	118.833	CBM35	PA14 ，CBM_6 ，DUF1080	Tat	Scaffold3_1
11	gnl|PRJNA209578|M768_15505	35.2	23	49.057	CBM6	ThuA	Sec	Scaffold6_1
12	gnl|PRJNA209578|M768_00810	19.2	19	126.632	GH2	PA14 ，Glyco_hydro_2_N ，Glyco_hydro_2 ，Glyco_hydro_2_C ，Laminin_G_3	Sec	Scaffold1_1
13	gnl|PRJNA209578|M768_01395	25.9	33	95.422	GH92	Glyco_hydro_92	Sec	Scaffold1_1

14	gnl|PRJNA209578|M768_08215	16	14	130.08	CE4 CBM9 CBM22 GH10	CBM_4_9 ， Polysacc_deac_1 ， Glyco_hydro_10 ， DUF1083	Sec	Scaffold2_1
15	gnl|PRJNA209578|M768_11570	33.5	22	40.345	AA1	Cu-oxidase_3 ， Cu-oxidase_2	Tat	Scaffold3_1
16	gnl|PRJNA209578|M768_00700	5.6	11	269.631	N.D.	——	Sec	Scaffold1_1
17	gnl|PRJNA209578|M768_16095	17.4	10	74.749	CBM6 GH92	PAP2	Tat	Scaffold7_1
18	gnl|PRJNA209578|M768_06610	2.7	5	198.125	CBM6	ThuA ， GSDH ， PKD ， CBM_6	Sec	Scaffold2_1
19	gnl|PRJNA209578|M768_13690	54.5	38	21.119	CE1	——	Sec	Scaffold5_1
20	gnl|PRJNA209578|M768_10165	19.1	6	38.576	CBM32	DUF1080	Sec	Scaffold3_1
21	gnl|PRJNA209578|M768_04525	3.2	3	134.483	GH51	CBM_4_9 ， Alpha-L-AF_C ， Laminin_G_3	Sec	Scaffold1_1
22	gnl|PRJNA209578|M768_02295	3.9	4	138.398	CBM32	F5_F8_type_C	Tat	Scaffold1_1
23	gnl|PRJNA209578|M768_10315	5.2	6	174.762	CBM6 GH92	Glyco_hydro_92 ， CBM_6	Sec	Scaffold3_1
24	gnl|PRJNA209578|M768_04540	6.2	3	84.49	CBM6 GH43 CBM22	CBM_4_9 ， Glyco_hydro_43 ， CBM_6	Tat	Scaffold1_1
25	gnl|PRJNA209578|M768_09190	12.8	6	58.944	CBM32 GH81	AP_endonuc_2 ， F5_F8_type_C	Sec	Scaffold2_1
26	gnl|PRJNA209578|M768_19580	11.2	2	30.654	N.D.	——	N.D.	Scaffold4_3
27	gnl|PRJNA209578|M768_04560	5.1	3	68.734	GH121	Big_4 ， Big_3_4	Tat	Scaffold1_1

Supplementary Information II

Predicted Secondary Structure of the Hypothetic Protein M768_06655

Sequence length	1642
Secondary structure	Helix=3.9%, Strand=31.2%, Loop=64.9%
window size	70
Structure content cutoff	12%
Minimum consecutive exposed residues	10
NORS	N=NORS region
Secondary structure	H=helix, E=strand, ' '=loop
Coiled-coil region	c=coils
Transmembrane helix	m=transmembrane helix
Solvent accessibility	e=exposed, ' '=buried
NORS region predicted:	398-472,482-621

```
              .    :    .    :    .    :    .    :    .    5
SEQ    MAAATSATAATVPAGVAAAPVVTAADTEPFDVLVFSKTGGFRHGSIPAGI
NORS
SEC       HHHHHHHHHHHHHH           EEEEEEE         HHHH
COILS
HTM
ACC    eee                 eeeeeee e     eee  e    e
              .    :    .    :    .    :    .    :    .    10
SEQ    AAIQKLGTENNFEVTATEDAGAFTDANLEQYDAVVWLSTTGDVLNDDQQA
NORS
SEC    HHHHHHHHH   EEEEE       HHHHH   EEEEEE      HHHHH
COILS
HTM
ACC    e  ee  eeee e e  ee ee  eee ee          e  eee ee
              .    :    .    :    .    :    .    :    .    15
```

```
SEQ    AFERYIQNGGGYAGIHAASDTEYDWPWYGGLVGAYFSAHPQNQTATVKVE
NORS
SEC    HHHHHHHH   EEEEE        HHHHHHH  EE       EEEEEE
COILS
HTM
ACC      ee  e            eee e             ee e e e e
           .    :    .    :    .    :    .    :    .   20
SEQ    DHVHESTAHLPSRWERFDEWYNFRTNPRDTVHVLASLDETTYNAGSGAMG
NORS
SEC                EEE   EEEEEE    EEEEEEE
COILS
HTM
ACC    eee    ee  ee e      e eeeeee       eeee eeeeeeee
           .    :    .    :    .    :    .    :    .   25
SEQ    AEHPTAWCQVYDGGRSWYTGGGHTDESYADPDFLQHLLGGIQTAAGAVAS
NORS
SEC        EEEEEE   EEEEEE         HHHHHHHHHHHHEEE
COILS
HTM
ACC    ee      e ee          ee  ee e  e         eeeee
           .    :    .    :    .    :    .    :    .   30
SEQ    DCAATQSASYEQIALDEETRNPMVLDVADDGTVFYAERDGRVQVIDPSTN
NORS
SEC              EEEEE      EEEE     EEEEE   EEEEEE
COILS
HTM
ACC    e eeeeeee ee e eee e       ee       ee e   eeeee
           .    :    .    :    .    :    .    :    .   35
SEQ    TTHTATTLPVTTANEDGLLGLVLDPAFSENGWVYTYWAPANVGSDGPHNR
NORS
```

```
SEC      EEEEEEEEEE    EEEEEE        EEEEEEEE        EE
COILS
HTM
ACC    eee     e  e  e          ee         eeeeeee
           .    :    .    :    .    :    .    :    .   40
SEQ    ISRFDFDPATGTIDQATEKKVLTVTTQRNTCCHAGGDMVFDEDGNLILAT
NORS                                              NNN
SEC    EEEEEEE          EEEEEEE          EEEE    EEEEEE
COILS
HTM
ACC      e e eeeeee eeeeee      eee           ee e
           .    :    .    :    .    :    .    :    .   45
SEQ    GDNTNPFESGGYSPHDERAGRQDYDAQRSSANTNDLRGKVLRIHPEADGT
NORS   NNNNNNNNNNNNNNNNNNNNNNNNNNNNNNNNNNNNNNNNNNNNNNNNNN
SEC                                       EEEEE
COILS
HTM
ACC      e eeeeeee eeeeeeeeeeeeeeee    ee e e      eeeee
           .    :    .    :    .    :    .    :    .   50
SEQ    YTIPEGNMFAPGTEKTRPEIFAMGFRNPFRIGVDPYSGNILVGDYGPDAG
NORS   NNNNNNNNNNNNNNNNNNNNNN        NNNNNNNNNNNNNNNNNNN
SEC                     EEEE      EEEE     EEEE
COILS
HTM
ACC        e    e eee e                eee e       e e
           .    :    .    :    .    :    .    :    .   55
SEQ    SASPTRGPGNTVEWNVVSEPGFYGWPYCTGANADYLKYNFATGQSGAAFN
NORS   NNNNNNNNNNNNNNNNNNNNNNNNNNNNNNNNNNNNNNNNNNNNNNNNNN
SEC                  EE
COILS
```

```
HTM
ACC    e     e  e      ee e     e eeeeeeeeeee e eee
           .    :    .    :    .    :    .    :    .   60
SEQ    CDAGPVNQSPNNTGLTQLPPAIEAEIWYNYAGNPDFPEIGGGGAPMGGPV
NORS   NNNNNNNNNNNNNNNNNNNNNNNNNNNNNNNNNNNNNNNNNNNNNNNNNN
SEC                          EEEE                   E
COILS
HTM
ACC       e  eee eeeeeee ee          eeee ee ee
           .    :    .    :    .    :    .    :    .   65
SEQ    YQFDPELDSDVQWPEYWDGKAFLGEWNQGKLYSIQLEGENRDDIVDINRV
NORS   NNNNNNNNNNNNNNNNNNNNN
SEC    EE                EEEEEEE  EEEEEEE
COILS
HTM
ACC     e eee eee e ee  e e      ee  e  e eeeeeee  e  ee
           .    :    .    :    .    :    .    :    .   70
SEQ    MPKIFDPSAGFDRAMDFDFGPDGALYVVDWGSGFGGDNDSSGVYKVNYVK
NORS
SEC                   EEE    EEEEEE           EEEEEEE
COILS
HTM
ACC      e  e e e ee   e  ee        e   eeeee    e e  e
           .    :    .    :    .    :    .    :    .   75
SEQ    GNPSPIARASADVTSGHAPLTVQFSSEGTRHPAGDELTLQWTFGDGSEPS
NORS
SEC          EEE         EEEEE           EEEEEE
COILS
HTM
ACC    ee e   e   eeeee     e  eeeee eeeee e     eeeee
```

```
           .    :    .    :    .    :    .    :    .    80
SEQ    TEANPVHTYTENGSYTAQLVATDAQGQTGVANVSVVVGNDAPTISITFPD
NORS
SEC          EEEE  EEEEEEEEE       EEEEEEEE    EEEEEE
COILS
HTM
ACC    ee e e   eee e  e   e eeeee          e       ee
           .    :    .    :    .    :    .    :    .    85
SEQ    NGGFFQWGDQVRYEIAVDDPDGEVDCSQVELSTSLGHDSHAHPMDVLGGC
NORS
SEC       EEE    EEEEEEEE        EEEEEEEEE
COILS
HTM
ACC    ee   e eee e e e e eeee e  e e            e  e
           .    :    .    :    .    :    .    :    .    90
SEQ    EGVFQTARDEGHGIESNIFWVIEASYTDDGGAAGAPLTANDLQVLQPKLL
NORS
SEC      EEE           EEEEEEEEEE
COILS
HTM
ACC    e   e e eeee           e eeee  ee e  ee e   ee e
           .    :    .    :    .    :    .    :    .    95
SEQ    QAEFFTSTGRLADSTSTGDPGVQRETTSDTAGGGQNIGYIEPGDWWAYDP
NORS
SEC      EEEE                           EEEE     EEE
COILS
HTM
ACC    e e   e ee eeeeeeee e eee eeee eeee e  e ee    e
           .    :    .    :    .    :    .    :    .    100
SEQ    ISLYGVDSIALRAASPSNATISIRWNAADGPEIGTVQVPGTGDWQVYQNV
```

```
NORS
SEC          EEEEEE      EEEEEE        EEEE       EEE
COILS
HTM
ACC     e e  e        ee   e e e eeeee       e e e eee
          .    :    .    :    .    :    .    :    .    105
SEQ    SSTLTDVPSGTGTLYFVLTSGQANVNWLEFDGRGVTDNERPTIDSFEVTP
NORS
SEC      EE       EEEEEEE      EEEEE
COILS
HTM
ACC    e e ee  ee        e e e e ee e eeeeeeeeee e  e
          .    :    .    :    .    :    .    :    .    110
SEQ    TTGTAPLKVTATATASDPEDDAITFAWDAGLGDGFVDGTDSFEVTYDQPG
NORS
SEC           EEEEEE        EEEEEE           EEEEE
COILS
HTM
ACC    e ee e e      eeeeee      e    e  e  ee e   e e
          .    :    .    :    .    :    .    :    .    115
SEQ    TYRLQVRASDERGAYSVEYTTVTVKASQTGPGMCFSGRSDDFLGTDLDEN
NORS
SEC     EEEEEEEE      EEEEEEEEE       EEE
COILS
HTM
ACC         e  eee e e        e ee        e ee e  e eee
          .    :    .    :    .    :    .    :    .    120
SEQ    RWSVLNRDQNLVVRDGNLVIPTTATDFYSTDNTTVPNMVLQDLPDGPFTA
NORS
SEC       EE    EEE    EEEEE              EEEEE    EEE
```

```
COILS
HTM
ACC    e e   ee e  ee          e             e  e
           .   :    .    :   .    :   .    :    .   125
SEQ    TAKLTLPARQQYQQAGLVIWEDQDNYAKMVLQGRSAAADPATRIFQFIRE
NORS
SEC    EEEEEE         EEEE      EEEEE          EEEEE
COILS
HTM
ACC           e e e       eeeee e     ee            ee
           .   :    .    :   .    :   .    :    .   130
SEQ    ENAAPNEVNESNSAALGAAYPDTVYVRFVSNGENLQAAYSADGIDFTTMP
NORS
SEC                           EEEEEE     EEEEEE     EEE
COILS
HTM
ACC    ee e ee eee                ee ee       e  e
           .   :    .    :   .    :   .    :    .   135
SEQ    QTKSIADLENAKIGLVSFKGNNTASPVIEAAFDWFSITPDDTASAPGPDD
NORS
SEC               EEEEE        EEEEEEEEE
COILS
HTM
ACC      e  e ee e      e e          e  e   ee e ee ee
           .   :    .    :   .    :   .    :    .   140
SEQ    EFDGTALDACRWSIVREDPTGYRVADGSLQIDTTPTDIYGTDNRPVPNIV
NORS
SEC            EEEEEE      EEEE  EEEEE              EE
COILS
HTM
```

```
ACC    e e    e  e e eee  e    e   e e    e   e e
           .    :    .    :    .    :    .    :    .    145
SEQ    LQDQPGEEWTIETVVDGSALDRQYQQGGLIVYGDDDNYVKLDYVVDNQAG
NORS
SEC    EE      EEEEEEEE          EEEEE     EEEEEEE
COILS
HTM
ACC      e  e e      e    ee  e       eeee  e e  eee  e
           .    :    .    :    .    :    .    :    .    150
SEQ    QAKNARIELRSEVAGVVQNPQPQSSSLTGDVWHLRLTRSGDTFTGEYSAD
NORS
SEC         EEEEE                   EEEEEEE     EEE
COILS
HTM
ACC    e e  e e   e e       e                e       e
           .    :    .    :    .    :    .    :    .    155
SEQ    GETWTTFDQSVTNAPASGAQVGLFALGASADTGAPASFGHFRVVGDEVEP
NORS
SEC                     EEEEEEE         EEEEEEE
COILS
HTM
ACC     ee    e       ee e       e e         e  eee e
           .    :    .    :    .    :    .    :    .    160
SEQ    IAVTITAETRCMAGKVFVAVRATNDDAVPVAITLETPYGSKEFADVAPGA
NORS
SEC                  EEEEEEE       EEEEEE    EEEEEE
COILS
HTM
ACC            e         e  eeee e       eeeee
           .    :    .    :    .    :    .    :    .    165
```

```
SEQ    NAYQSFASRAVGIDAGSVTVTATDAEGRTFSGTADYAARACS
NORS
SEC      EEE          EEEEEEE          EEEEEE
COILS
HTM
ACC    e      e  eeee        eeeee e       eeee
//
```

Predicted by: Reference:

1. PredictProtein: B Rost,G Yachdav and J Liu (2004) The PredictProtein Server. Nucleic Acids Research 32(Web Server issue):W321-W326.
    - Author: B Rost
    - Contact: help@predictprotein.org
    - URL: PredictProtein Homepage
    - Description: PredictProtein is the acronym for all prediction programs run.
2. PROFphd: B Rost (1996) Methods in Enzymology, 266:525-539
    - Author: B Rost
    - Description: PROF is a suite of programs predicting 1D structure (secondary structure, solvent accessibility) from multiple sequence alignments.PHDhtm predicts the location and topology of transmembrane helices from multiple sequence alignments.
    - URL: PROFphd Information Page

3. NORS: Liu J, Rost B (2003) NORSp: predictions of long regions without regular secondary structure. Nucleic Acids Research 31(13):3833-3835
    - Author: J. Liu
    - URL: NORSp Information Page
    - Description: NORSp is a predictor of NOn-Regular Secondary Structure.
